# Supplementary material for: Reconciling Mining with the Conservation of Cave Biodiversity: A Quantitative Baseline to Help Establish Conservation Priorities
Source: PLoS One. 2016 Dec 20;11(12):e0168348. doi: 10.1371/journal.pone.0168348 (PMC5173368; doi:10.1371/journal.pone.0168348)
Supplement: S1 Dataset — (ZIP) [file pone.0168348.s002.zip › Taxa/Serra Norte/SN_2013/N5S/Lista N5S-69.pdf]

CAVIDADE N5S-0069

Classe	Ordem	Fam/Outros	Gên/Outros	Espécie	S-U	U-S	Zon.
Annelida	Oligochaeta			jovem		2	E
Arachnida	Acari	Veigaiidae		sp.1	X		E
Arachnida	Amblypygi	Phrynidae	*Heterophrynus*	*longicornis*	1	3	E
Arachnida	Araneae	Araneidae		jovem	X		E
Arachnida	Araneae	Ctenidae	*Phoneutria*	*reidyi*		1	E
Arachnida	Araneae	Mysmenidae	*Maymena*	sp.1	X		E
Arachnida	Araneae	Oonopidae		jovem	X	X	E
Arachnida	Araneae	Pholcidae		jovem	X		E
Arachnida	Araneae	Pisauridae	*Architis*	sp.1		2	E
Arachnida	Araneae	Pisauridae	*Thaumasia*	*velox*		2	E
Arachnida	Araneae	Salticidae		sp.7	X	X	E
Arachnida	Araneae	Theraphosidae		jovem	2	1	E
Arachnida	Araneae	Theridiidae		jovem		X	E
Arachnida	Araneae	Theridiosomatidae	*Plato*	sp.1	X		E
Arachnida	Araneae	Theridiosomatidae	*Plato*	sp.1	X	X	E
Arachnida	Araneae	Trechaleidae	*Rhoicinus*	sp.1	2		E
Arachnida	Opiliones	Cosmetidae	*Roquettea*	*singularis*		2	E
Arachnida	Opiliones	Sclerosomatidae	*Prionostema*	sp.1		2	E
Arachnida	Opiliones	Stygnidae	*Protimesius*	*gracilis*		1	E
Chilopoda	Scolopendromorpha	Cryptopidae	*Cryptops*	jovem	1		E
Diplopoda	Polydesmida	Pyrgodesmidae		jovem		X	E
Entognatha	Collembola	Cyphoderidae		sp.1		X	E
Entognatha	Collembola	Entomobryidae		sp.1	X		E
Entognatha	Collembola	Paronellidae		sp.1		X	E
Entognatha	Collembola	Paronellidae		sp.4	X		E
Entognatha	Diplura	Campodeidae		sp.1		X	E
Insecta	Blattodea			jovem	1	1	E
Insecta	Coleoptera	Cerylonidae		sp.1		X	E
Insecta	Coleoptera	Hydrophilidae		sp.2	X		E
Insecta	Coleoptera	Scydmaenidae		sp.2	X		E
Insecta	Coleoptera	Staphylinidae	Pselaphinae	sp.4	X		E
Insecta	Coleoptera	Staphylinidae		sp.4	X		E
Insecta	Diptera	Cecidomyiidae	Cecidomyiinae	sp.		X	E
Insecta	Diptera	Tipulidae	Tipulinae	sp.	X	X	E
Insecta	Ephemeroptera	Leptophlebiidae		jovem		X	E
Insecta	Heteroptera	Dipsocoroidea		sp.	X		E
Insecta	Heteroptera	Reduviidae	Reduviinae	sp.	2		E
Insecta	Heteroptera	Veliidae	*Microvelia*	jovem		X	E
Insecta	Homoptera	Cixiidae		sp.3		X	E
Insecta	Hymenoptera	Formicidae	*Camponotus*	sp.1		X	E
Insecta	Hymenoptera	Formicidae	*Crematogaster*	sp.1	X		E
Insecta	Hymenoptera	Formicidae	*Hypoponera*	sp.1		X	E
Insecta	Hymenoptera	Formicidae	*Paratrechina*	sp.1	X	X	E
Insecta	Hymenoptera	Formicidae	*Paratrechina*	sp.2	X		E
Insecta	Hymenoptera	Formicidae	*Pheidole*	sp.3	X	X	E
Insecta	Hymenoptera	Formicidae	*Solenopsis*	sp.2		X	E
Insecta	Hymenoptera	Formicidae	*Trachymyrmex*	sp.1	X		E
Insecta	Isoptera	Termitidae	*Nasutitermes*	sp.	X	X	E
Insecta	Lepidoptera	Gelechioidea		sp.	X		E

Insecta	Orthoptera	Phalangopsidae	*Paraclodes*	sp.	9	8	E
Insecta	Orthoptera	Phalangopsidae	*Phalangopsis*	sp.		1	E
Insecta	Thysanura	Nicoletiidae		sp.1	X	X	E
Gastropoda	Pulmonata	Systrophiidae	*Happia*	sp.		X	E
Malacostraca	Isopoda	Philosciidae		sp.1		X	E
Turbellaria				sp.4	X		E
Amphibia	Anura	Strabomantidae	*Pristimantis*	cf. *fenestratus*	5	2	E
Mammalia	Chiroptera	Emballonuridae	*Peropteryx*	*macrotis*		1	E
Reptilia	Squamata	Sauria		sp.	1		E
